# Photoinduced Zn‐Air Battery‐Assisted Self‐Powered Sensor Utilizing Cobalt and Sulfur Co‐Doped Carbon Nitride for Portable Detection Device

**DOI:** 10.1002/advs.202408293

**Published:** 2024-10-24

**Authors:** Yun Chen, Yuhang Ge, Yuting Yan, Li Xu, Xingwang Zhu, Pengcheng Yan, Penghui Ding, Huaming Li, Henan Li

**Affiliations:** ^1^ School of Chemistry and Chemical Engineering Institute for Energy Research School of Agricultural Engineering Jiangsu University Zhenjiang 212013 China; ^2^ School of Environmental Science and Engineering College of Mechanical Engineering Yangzhou University Yangzhou 225002 China; ^3^ Department of Science and Technology Linköping University Norrköping SE‐601 74 Sweden

**Keywords:** carbon nitride, device, portability, self‐powered sensor, Zn‐air battery

## Abstract

Most self‐powered electrochemical sensors (SPESs) are limited by low open circuit voltage and power density, leading to a narrow detection range and low sensitivity. Herein, a photoinduced Zn‐air battery‐assisted SPES (ZAB‐SPES) is proposed based on cobalt and sulfur co‐doped carbon nitride with the cyano group (Co, S‐CN). The cyano functionalization remarkably enhances visible light utilization, and the cyano moiety acts as an electron‐withdrawing group to promote electron enrichment. Co and S co‐doping can create a *p–n* homojunction within carbon nitride, enabling the efficient migration and separation of carriers, thereby significantly improving the performance of the oxygen reduction reaction. The synergistic effects endow Co, S‐CN photocathode with an open circuit voltage of 1.85 V and the maximum power density of 43.5 µW cm^−2^ in the photoinduced ZAB. Employing heavy metal copper ions as the target model, the photoinduced ZAB‐SPES exhibited dual‐mode and sensitive detection. Furthermore, a portable detection device based on the photoinduced ZAB‐SPES is designed and exhibits high linearity in the range of 5 ~ 600 nm with a detection limit of 1.7 nm. This work offers a portable detection method based on the photoinduced ZAB‐SPES in the aquatic environment.

## Graphical abstract

A photoinduced Zn‐air battery‐assisted self‐powered electrochemical sensor (ZAB‐SPES) is proposed based on cobalt and sulfur co‐doped carbon nitride with the cyano group (Co, S‐CN). Co, S‐CN photocathode possesses excellent performance of the photoinduced ZAB with an open circuit voltage of 1.85 V. A device is designed for achieving portable and on‐site detection of Cu(II) in the aquatic environment.

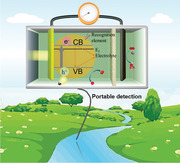

## 1 Introduction

Self‐powered electrochemical sensors (SPESs) harness energy from the environment, eliminating the need for batteries or external power sources. SPESs are extensively applied for detecting various substances including biological small molecules, tumor cells, enzyme catalysis, and environmental pollutants.^[^ ^1^, ^2^ ^]^ As an emerging detection platform, it holds considerable promise in wearable, implantable, miniaturized, and portable energy devices. The output electrical signals, such as open circuit voltage (E_ocv_) or power density, are directly influenced by the interaction between the target and the electrode. However, most SPESs exhibit several limitations, including low E_ocv_ (< 0.6 V), low power density, and inefficient utilization of environmental energy sources (such as substrate and light). These limitations result in a narrow detection range and low detection sensitivity, hindering the widespread application and development of SPESs.^[^ ^3^ ^]^ Thus, designing SPESs with high E_ocv_ or power density is crucial for sensitive detection of targets.

Photoinduced zinc‐air batteries (ZABs) can convert both chemical and light energy into electrical energy, enhancing the actual E_ocv_ to 1.78 V.^[^ ^4^, ^5^, ^6^ ^]^ The photoinduced ZABs utilize Zn and a photocatalyst as the anode and photocathode for Zn oxidation and oxygen reduction reactions (ORR), respectively.^[^ ^7^, ^8^, ^9^ ^]^ The integration of the photoinduced ZAB into the SPES (ZAB‐SPES) is expected to enhance the E_ocv_ and power density of the SPES, thereby improving the sensitivity and widening the linear detection range for the target analytes.^[^ ^3^, ^10^, ^11^, ^12^ ^]^ The working principle of the photoinduced ZAB‐SPES is as follows: the photocathode material generates electrons and holes when excited by light, and the electrons produced by the catalytic oxidation reaction of Zn anode are transferred to the photocathode, generating E_ocv_ and power density. Electrons in the conduction band (CB) of the photocathode, along with that from the Zn oxidation, drive the ORR. When the photocathode is covered by the target analyte, it hinders visible light capture and increases steric hindrance to suppress electron transfer, resulting in sluggish ORR, and thus  decreasing the output signals.^[^ ^3^, ^13^, ^14^, ^15^ ^]^ Wang et al. utilized poly(1,4‐di(2‐thiophenyl)benzene (PDTB) as a photocathode to construct a photoinduced ZAB‐SPES for detecting miRNA let‐7a.^[^ ^10^ ^]^ Through competition between PDTB and the metal–organic framework with glucose oxidase redox, the output power density of ZAB was reduced, and the self‐powered biosensor achieved ultra‐sensitive detection of miRNA let‐7a with a detection limit as low as 1.38 fM.

Designing photoactive materials as the photocathodes of the photoinduced ZABs to widen the light utilization and accelerate electron transfer for ORR is a pivotal aspect in constructing the SPESs. The theoretical E_ocv_ value of a photoinduced ZAB‐SPES is defined by the redox potential of Zn/Zn^2+^ and the quasi‐Fermi level of the photocathode.^[^ ^16^, ^17^, ^18^ ^]^ Thus, the band structure of the semiconductor serving as the photocathode significantly affects the ZABs performance for constructing a highly sensitive SPES. The development of a photocathode should focus on the following factors: the CB potential is lower than that of the reduction of O_2_ to H_2_O (0.4 V vs NHE, pH 7) to drive ORR and higher than that of the reduction of Zn/Zn^2+^ (−1.25 V vs NHE) to inhibit Zn^2+^ reduction.^[^ ^8^ ^]^ Graphitic carbon nitride (g‐C_3_N_4_) has been reported to possess the highest and lowest occupied orbitals, located at 1.4 and −1.3 V (vs NHE, pH 7),^[^ ^19^, ^20^, ^21^, ^22^, ^23^, ^24^, ^25^, ^26^ ^]^ meeting the thermodynamic requirement for ORR in the photoinduced ZABs. However, due to the negative CB potential to Zn/Zn^2+^ reduction potential and low visible light utilization, the electronic structure is optimized by introducing molecular control and structural design strategies,^[^ ^27^, ^28^, ^29^ ^]^ such as element doping, defect engineering, and functional group modification, which satisfies the CB potential between the Zn/Zn^2+^ and O_2_/H_2_O reduction potential. Shen et al. proposed cyano‐functionalized crystalline g‐C_3_N_4_ by KSCN molten salts to broaden visible light utilization, whereby the cyano acts as an electron‐absorbing group to promote electron accumulation and inhibit recombination.^[^ ^30^, ^31^ ^]^ An internal electric field can be formed by constructing a g‐C_3_N_4_‐based homojunction to achieve carrier separation and transfer,^[^ ^32^, ^33^, ^34^, ^35^ ^]^ enhancing the ORR performance by the photoinduced electrons. This study aims to enhance the photocatalytic ORR performance by element doping, functional group modification, and homojunction construction, thereby amplifying E_ocv_ and power density of the photoinduced ZAB.

In this work, we develops a photoinduced ZAB‐SPES utilizing cobalt and sulfur co‐doped g‐C_3_N_4_ with the cyano group (Co, S‐CN) as a photocathode. The synergistic effects of the cyano functionalization and Co,S co‐doping on visible light harvesting ability and carrier separation/transport efficiency are investigated to enhance the photocatalytic ORR performance. The changes in E_ocv_ and power density of the photoinduced ZAB based on Co, S‐CN photocathode are evaluated. With copper ions (Cu(II)) as the target model, the photoinduced ZAB‐SPES is capable of dual‐mode detection. A portable detection device based on the photoinduced ZAB‐SPES is designed with some components, and the feasibility is explored to improve the portable determination of Cu(II) in the water environment.

## 2 Results and Discussion

### 2.1 Morphological and Structural Characterization

**Figure 1:**
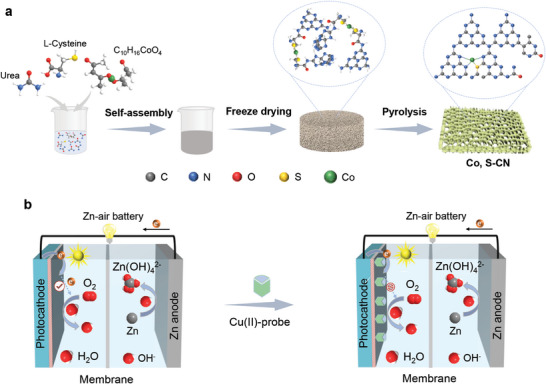
a) Synthetic diagram of Co, S‐CN by self‐assembly and pyrolysis. b) Schematic illustration of the photoinduced ZAB‐SPES for Cu(II) detection.

Co, S‐CN was synthesized by self‐assembly and pyrolysis of three organic molecules among urea, L‐Cysteine (L‐Cys), and C_10_H_16_CoO_4_ (**Figure** **1**a; Scheme S1c, Supporting Information). L‐Cys contains the carboxyl (─COOH), amino (─NH_2_), and thiol (─SH) groups. The ─COOH from L‐Cys was converted to C═O through amination with urea, and the C═O was incorporated into the heptazine units.^[^ ^29^, ^36^ ^]^ L‐Cys forms insoluble mercaptide with Co^2+^ metal ions, referred to S─Co─S, which coordinated with heptazine ring through Co─N and Co─S to achieve sulfur doping. In situ keto‐enol cyclization occurred between C_10_H_16_CoO_4_ and urea, leading to the formation of 4,6‐dimethyl‐2‐hydroxypyrimidine (DHPD) in heptazine units.^[^ ^37^ ^]^ During the synthesis process, the cyano (C≡N) group was introduced by KHCO_3_ as a molten salt. Through the assembly and copolymerization of urea and L‐Cys, ─C═C─C─ of L‐Cys was introduced into the tri‐s‐triazine ring, replacing nitrogen atoms of C═N─C to form a C_4_N_2_ ring,^[^ ^38^ ^]^ thus generating carbon‐bridged g‐C_3_N_4_ with the C≡N group (CCN, Scheme S1b, Supporting Information).

**Figure 2:**
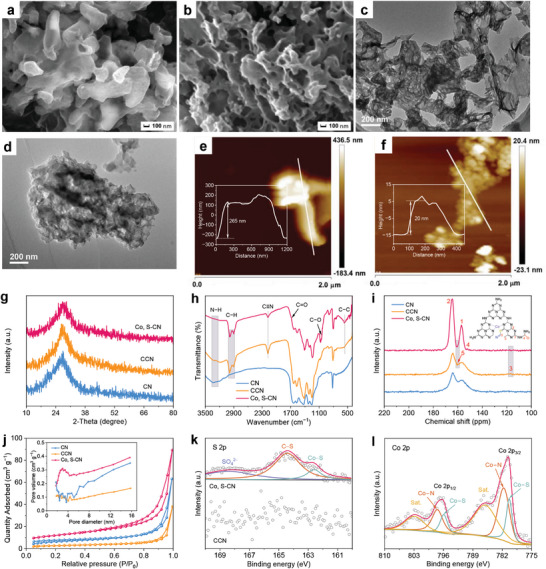
Morphology and structural characterization. SEM images of a) CN and b) Co, S‐CN. TEM images of c) CN and d) Co, S‐CN. AFM images and corresponding height of e) CN and f) Co, S‐CN. g) XRD patterns, h) ATR‐IR spectra, i) inset: Co, S‐CN structure) ^13^C NMR spectra, and j) inset: Pore size distribution) N_2_ adsorption‐desorption isotherms of CN, CCN, and Co, S‐CN. k) S 2p XPS spectra of CCN and Co, S‐CN. l) Co 2p XPS spectrum of Co, S‐CN.

From scanning electron microscope (SEM), transmission electron microscope (TEM), and atomic force microscopy (AFM) images, CN represents a hollow tubular morphology with porous structure (**Figure** **2**a,c,e; Figures S1a and S3a, Supporting Information). KHCO_3_ as the molten salt significantly affects the morphology, resulting in g‐C_3_N_4_ with the C≡N group (CN─C), representing a stacked bulk structure (Figure S2, Supporting Information). After the introduction of L‐Cys, the tubular is disrupted (Figures S1b,c and S3b,c, Supporting Information). In contrast to CN and CCN, Co, S‐CN exhibits thin and porous nanosheets morphology (Figure 2b,d; Figure S1d, Supporting Information), with the nanosheets appearing smaller (Figure S3d, Supporting Information). The height of Co, S‐CN nanosheets is ≈20 nm (Figure 2f), which is thinner than CCN (≈205 nm, Figure S4, Supporting Information). No nanoparticles or clusters are observed on the surface of the nanosheets. The thinner nanosheets can shorten the migration path of the photoinduced carrier and increase the specific surface area,^[^ ^39^ ^]^ and the porous structure increases the exposure of more active sites, facilitating oxygen adsorption for ORR to enhance the photoinduced ZAB performance. Energy‐dispersive X‐ray spectroscopy (EDX, Figure S6, Supporting Information) mapping images confirm the presence of C, N, O, Co, and S elements in Co, S‐CN. The zeta potential of CN was measured to be −12.5 mV, while CCN and Co, S‐CN show more negative zeta potentials (Figure S7, Supporting Information, −42.6 and −34.1 mV, respectively), attributed to the presence of OH^−^ on the CN surface. The incorporation of ─C═C─C─ renders CN with more negative charges, revealing more stable colloidal solution of CN‐C.^[^ ^40^ ^]^

X‐ray diffraction (XRD, Figure 2g) patterns distinctly reveal a broad peak centered at 27.4°, corresponding to the interlayer stacking (002) plane in the heptazine ring. Attenuated total reflectance infrared spectroscopy (ATR‐IR, Figure 2h) was conducted to confirm the incorporation of ─C═C─C─, C≡N, and C═O groups on the heptazine structure. The broad peak in the range of 3000 to 3400 cm^−1^ corresponds to the stretching vibration of terminal amino (N─H) groups. For CCN and Co, S‐CN, the N─H stretching vibration peak almost disappears, while new characteristic peaks emerge at 2900 ~ 3000, 2178, and 560 cm^−1^, corresponding to C─H stretching vibration,^[^ ^41^ ^]^ C≡N,^[^ ^42^, ^43^ ^]^ and C─C characteristics, respectively. The heat treatment assisted with KHCO_3_ molten salt facilitates the reaction between OH^−^ of KHCO_3_ and ─NH_2_ of heptazine units, reducing the amino content and forming C≡N,^[^ ^42^, ^43^, ^44^ ^]^ thus generating C─H stretching vibration. C─C bonds in CCN originate from the ─C═C─C─ chain of L‐Cys. Co, S‐CN exhibits a characteristic peak of C─O (1068 cm^−1^), attributed to the formation of DHPD through cyclization between C_10_H_16_CoO_4_ and urea.^[^ ^45^ ^]^ A weak absorption peak corresponding to the C═O vibration (1690 cm^−1^) is observed in Co, S‐CN, and urea undergoed a condensation reaction to generate melamine, which interacted with L‐Cys via amination to convert ─COOH to C═O.^[^ ^29^ ^]^

Solid ^13^C nuclear magnetic resonance (NMR, Figure 2i) spectra reveal two prominent peaks at 156.6 and 164.1 ppm, corresponding to CN_3_ (C1) and CN_2_−NH_x_ (C2) in melem units. The intensity ratio of C1/C2 changes in the presence of ─C─C═C─, C≡N, and C═O.^[^ ^46^ ^]^ New peaks at 116.9 and 159.9 ppm in CCN are assigned to ─C≡N (C3) and ─C─C═C─ (C5),^[^ ^36^, ^47^ ^]^ consistent with infrared results. In Co, S‐CN, a peak located at 151.8 ppm belongs to CN_2═_O (C4) of L‐Cys. Melamine was generated by urea condensation, while Co^2+^ and L‐Cys reacted and further self‐assembled with melamine to form the C═O. All prepared samples exhibit type‐IV absorption–desorption isotherms with a mesoporous structure (Figure 2j). The specific surface area (S_BET_) of CN, CCN, and Co, S‐CN were determined to be 22.71, 9.64, and 41.68 m^2^ g^−1^, respectively (Table S1, Supporting Information). CN exhibits a hollow tubular shape with a large S_BET_, providing the basis for modification. The thin nanosheet‐like structure of Co, S‐CN increases the surface area, and the porous volume enables the exposure of more active sites, enhancing oxygen absorption ability, improving ORR performance, and thus increasing the E_ocv_ and the maximum power density (P_max_) of the photoinduced ZAB. The C/N atomic ratio of CN, CCN, and Co, S‐CN were determined as 0.549, 0.605, and 0.613 from organic element analysis (Table S2, Supporting Information). The loading content of Co element in Co, S‐CN was to be 0.60 wt% by inductively coupled plasma mass spectrometry (ICP‐MS, Table S3, Supporting Information).

Survey X‐ray photoelectron spectroscopy (XPS) spectra exhibit Co, O, N, K, C, and S signals in Co, S‐CN (Figure S9, Supporting Information). In the C 1s XPS spectra (Figure S10a, Supporting Information), two typical peaks at 287.9 and 284.8 eV correspond to N─C═N and C─C/C═C within heptazine units. Upon the introduction of molten salts, a new peak emerges at 286.0 eV, attributed to C≡N. For Co, S‐CN, two peaks appear at 289.2 and 286.7 eV, assigned to C═O of L‐Cys and C−O of DHPD, respectively.^[^ ^48^, ^49^ ^]^ The area ratio of C─C/C═C to N─C═N in CCN and Co, S‐CN increases to 0.379 and 0.290 from the C 1s XPS spectra (Table S4, Supporting Information). Both CCN and Co, S‐CN have negative shifts in the binding energies of C 1s and N 1s (Figure S10b, Supporting Information) XPS spectra. Regarding O 1s spectra (Figure S10c, Supporting Information), a single peak corresponding to O─H from absorbed water molecules is observed. In the Co, S‐CN, a characteristic peak at 530.5 eV signifies the presence of C═O bonds. CCN exhibits clear noise of S 2p XPS spectrum (Figure 2k), indicating H_2_S gas production from the ─SH of L‐Cys during high‐temperature pyrolysis, with S unable to be doped into the heptazine units.^[^ ^36^ ^]^ For Co, S‐CN, distinct peaks at 168.4, 164.4, and 162.6 eV are fitted, attributed to sulfate species (SO_4_^2−^), C─S, and Co─S bonds, respectively.^[^ ^50^, ^51^ ^]^ In the Co 2p spectrum (Figure 2l), Co, S‐CN exhibits Co 2p_1/2_ and Co 2p_3/2_ orbits fitted with Co─N and Co─S coordination.^[^ ^51^ ^]^ Two peaks at 781.5 and 797.3 eV are assigned to Co^2+^, while peaks at 780.2 and 795.8 eV correspond to Co^3+^.^[^ ^52^, ^53^ ^]^ −COOH undergoes conversion to C═O during amination, and the formation of Co─S by complexation reaction leads to S doped into heptazine units.

**Figure 3:**
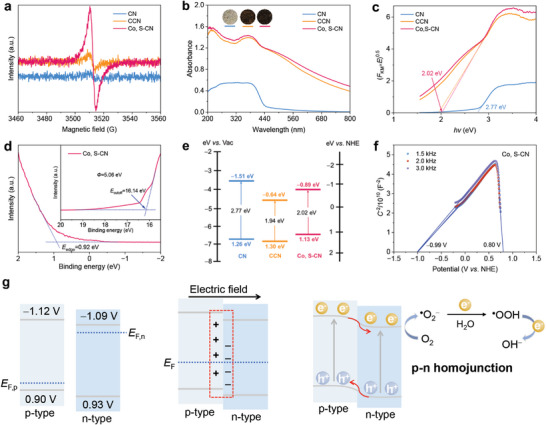
Optical property and electronic band structure. a) EPR spectra, b) inset: The color of these samples) UV–Vis absorption spectra, c) Tauc plots, and e) band structure alignment of CN, CCN, and Co, S‐CN. d) UPS spectrum and f) Mott–Schottky plots of Co, S‐CN. g) Schematic diagram of charge transfer path in Co, S‐CN homojunction.

The presence of the defects was confirmed by electron paramagnetic resonance spectroscopy (EPR, **Figure** **3**a). EPR signal intensity of CCN exceeds that of CN, and Co, S‐CN represents the strongest signal. The incorporation of ─C─C═C─ from L‐Cys into CCN leads to the replacement of nitrogen atoms with carbon atoms in the heptazine units, and the introduction of molten salts promotes the formation of C≡N, thus generating the defects. The introduction of L‐Cys and Co(II) salts caused color changes from white to brown and dark brown for CCN and Co, S‐CN, respectively (inset Figure 3b). Notably, Co, S‐CN exhibits enhanced light absorption, particularly in the visible region, with the maximum absorption edge broadening from 455 to 720 nm (Figure 3b). Co, S‐CN has superior efficiency in sunlight absorption, facilitating the generation of charge carriers, which lays the foundation for charge separation and migration, thus increasing the output response signals of the photoinduced ZAB. As depicted in Figure 3c, the bandgaps (*E*_g_) as derived from Tauc plots were determined to be 2.77, 1.94, and 2.02 eV for CN, CCN, and Co, S‐CN, respectively, which align with the observed redshift in light absorption. The electronic band structure was elucidated through UV–Vis absorption spectra and ultraviolet photoelectron spectroscopy (UPS, Figure 3d; Figure S12, Supporting Information). The energy of the valence band (*E*_VB_ vs vac, *E*_vac_) for CN, CCN, and Co, S‐CN was calculated to be −6.11, −6.15, and −5.98 eV (vs vac) using the equation *hv* + *E*_edge_ − *E*_cutoff_, where *hv* denotes the excitation energy of the He I source (21.2 eV).^[^ ^54^, ^55^, ^56^ ^]^ *E*_VB_ values (vs NHE, *E*_NHE_) were determined using the equation *E*_vac_ = −*E*_NHE_ − 0.059 pH − 4.44 eV. Complemented by *E*_g_ values from Tauc plots, the energy of the conduction band minimum (*E*_CB_) and the Fermi level (*E*_F_) are detailed in Table S5 (Supporting Information), with the electronic band structure of CN, CCN, and Co, S‐CN illustrated in Figure 3e. Mott–Schottky plots reveal that CN and CN‐C are n‐type semiconductors (Figure S13, Supporting Information), while Co, S‐CN has both n‐type and p‐type domains (Figure 3f). L‐Cys as a carbon source does not alter the n‐type region. The Fermi level of n‐type semiconductors (*E*_F,n_) closely aligns with the *E*_CB_, whereas that of p‐type semiconductors (*E*_F,p_) approaches the *E*_VB_. The band positions of n‐type and p‐type fragments were determined accordingly. To achieve Fermi level equilibrium, a new internal electric field formed between n‐type and p‐type regions, facilitating the migration of photoinduced electrons from p‐type to n‐type domains and holes from n‐type to p‐type. Co and S co‐doping is beneficial to the formation of a *p–n* homojunction in Co, S‐CN, fostering the efficient migration of photoinduced electrons, which enhances the oxygen reduction to generate the superoxide radicals (^•^O_2_^−^), peroxide hydroxyl radicals (^•^OOH), and ultimately OH^−^, thereby improving the performance of the photoinduced ZAB. The band structure, the construction of the electric field, and a *p–n* homojunction in Co, S‐CN are illustrated in Figure 3g.

### 2.2 Photoelectrochemical Performance

**Figure 4:**
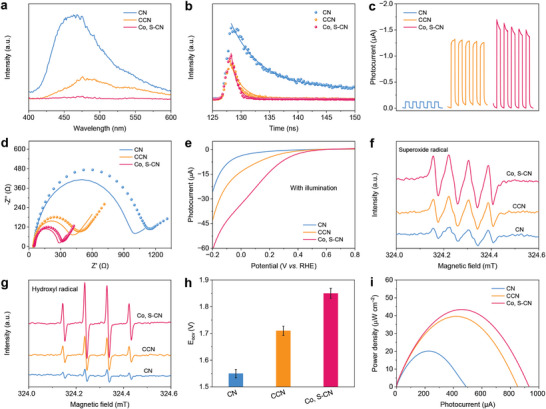
Photoelectrochemical properties. a) Steady‐state FL spectra, b) transient FL spectra, c) *I–t* curves, d) inset: The dashed and solid lines represent EIS spectra without and with light irradiation) EIS spectra, and e) LSV curves of CN, CCN, and Co, S‐CN. ESR spectra of f) DMPO‐superoxide and g) DMPO‐hydroxyl radicals with illumination for 5 min in CN, CCN, and Co, S‐CN. h) E_ocv_ values and i) *P–I* curves of the photoinduced ZAB for CN, CCN, and Co, S‐CN. All error bars were obtained by three measurements.

In contrast to CN and CCN, Co, S‐CN shows the lowest signal intensity in steady‐state fluorescence (FL) spectra (**Figure** **4**a). The emission peaks of all samples are redshifted, which well aligns with the results from the UV–Vis absorption spectra. Based on the transient FL spectra, the average lifetime (*τ*_av_) values for CN, CCN, and Co, S‐CN were calculated to be 5.26, 2.02, and 1.03 ns, respectively (Figure 4b; Table S6, Supporting Information). The decrease in carrier lifespan and emission intensity demonstrates that Co, S‐CN possesses a strong ability to absorb and activate O_2_ due to efficient excitation, transport, and separation of charge carriers. These prepared samples were modified on indium tin oxide (ITO) electrodes, and transient photocurrent–time (*I–t*) curves were obtained under 20 s of on‐off light irradiation without a bias voltage. The photocurrent responses of CN/ITO, CCN/ITO, and Co, S‐CN/ITO photocathodes were −0.12, −1.22, and −1.40 µA, respectively (Figure 4c). The arc radius of all samples under light irradiation was smaller than that in dark conditions, implying fast charge transport after light excitation (Figure 4d). Co, S‐CN/ITO had the highest photocurrent and the smallest arc radius, indicating the most effective carrier separation and charge transfer. The content of L‐Cys and Co salts during the synthesis and the fixed area of Co, S‐CN during the modification were optimized (Figures S14–S16, Supporting Information).

Linear cyclic voltammetry (LSV) curves for CN/ITO, CCN/ITO, and Co, S‐CN/ITO were recorded to assess the ORR performance under continuous light illumination in phosphate buffer (PB, 0.1 m, pH 7.0) containing the saturated O_2_. Figure 4e illustrates that Co, S‐CN/ITO had a high initial potential, while the decline in current was remarkable. As shown in Figure S17 (Supporting Information), a low current signal for Co, S‐CN was observed under saturated N_2_ conditions. Co, S‐CN exhibited superior ORR performance, indicating the potential for designing and constructing a photoinduced ZAB‐SPES as the photocathode. The thin nanosheets can shorten the carrier transport path, and the porous structure promotes oxygen transmission. The C≡N modification in the heptazine units remarkably enhances sunlight utilization and serves as an electron‐withdrawing group to promote electron enrichment and inhibit recombination. An electric field is generated to form an internal *p–n* homojunction, facilitating electron separation and migration. Also, Co doping provides more active sites, thus improving the ORR performance. The reaction species of superoxide radical (^•^O_2_^−^) and hydroxyl radical (^•^OH) in photocatalytic ORR were examined using electron spin resonance (ESR) with 5,5‐dimethyl‐1‐pyrroline N‐oxide (DMPO) as the solvent. ESR signals for all samples are not detected in the dark, and the intensity increases with prolonged light exposure (Figures S18 and S19, Supporting Information). Co, S‐CN exhibits the strongest signals for DMPO‐^•^O_2_^−^ and DMPO‐^•^OH (Figure 4f,g), indicating the highest electron concentration in the CB for reducing O_2_ to generate ^•^O_2_^−^. The built‐in electric field forms a *p–n* homojunction, efficiently separating electrons and holes, resulting in the high concentration of holes in the VB.

### 2.3 Detection Performance of the Photoinduced ZAB‐SPES

A photoinduced ZAB‐SPES was constructed using a Zn plate as the anode and the modified ITO as the photocathode, separated by a polyphenylene sulfide (PPS) membrane. E_ocv_‐time and voltage–photocurrent (*V–I*) curves were recorded with a Zn anode immersed in alkaline KOH (0.1 m) and the modified photocathode placed in PB (0.1 m) under xenon lamp illumination. Following light exposure, the E_ocv_ for the ZAB based on CN/ITO, CCN/ITO, and Co, S‐CN/ITO reached 1.55, 1.71, and 1.85 V, respectively (Figure 4h; Table S7, Supporting Information), exceeding the actual E_ocv_ of conventional ZABs (1.38 V). The photoinduced ZAB can convert both chemical and light energy into electrical energy, thereby enhancing the E_ocv_ and P_max_.^[^ ^9^, ^18^ ^]^ *P–I* curves, derived from the *V–I* curves using the formula P = V × I/A, yielded P_max_ of 20.1 µW cm^−2^ for CN, 33.0 µW cm^−2^ for CCN, and 43.5 µW cm^−2^ for Co, S‐CN (Figure 4i). The E_ocv_ of the ZAB system based on Co, S‐CN under light illumination was significantly higher than that measured in darkness (≈1.50 V, Figure S20, Supporting Information). The notable enhancement in E_ocv_ and P_max_ is attributed to the highly photocatalytic ORR performance of the Co, S‐CN photocathode, which amplifies the output signals.

**Figure 5:**
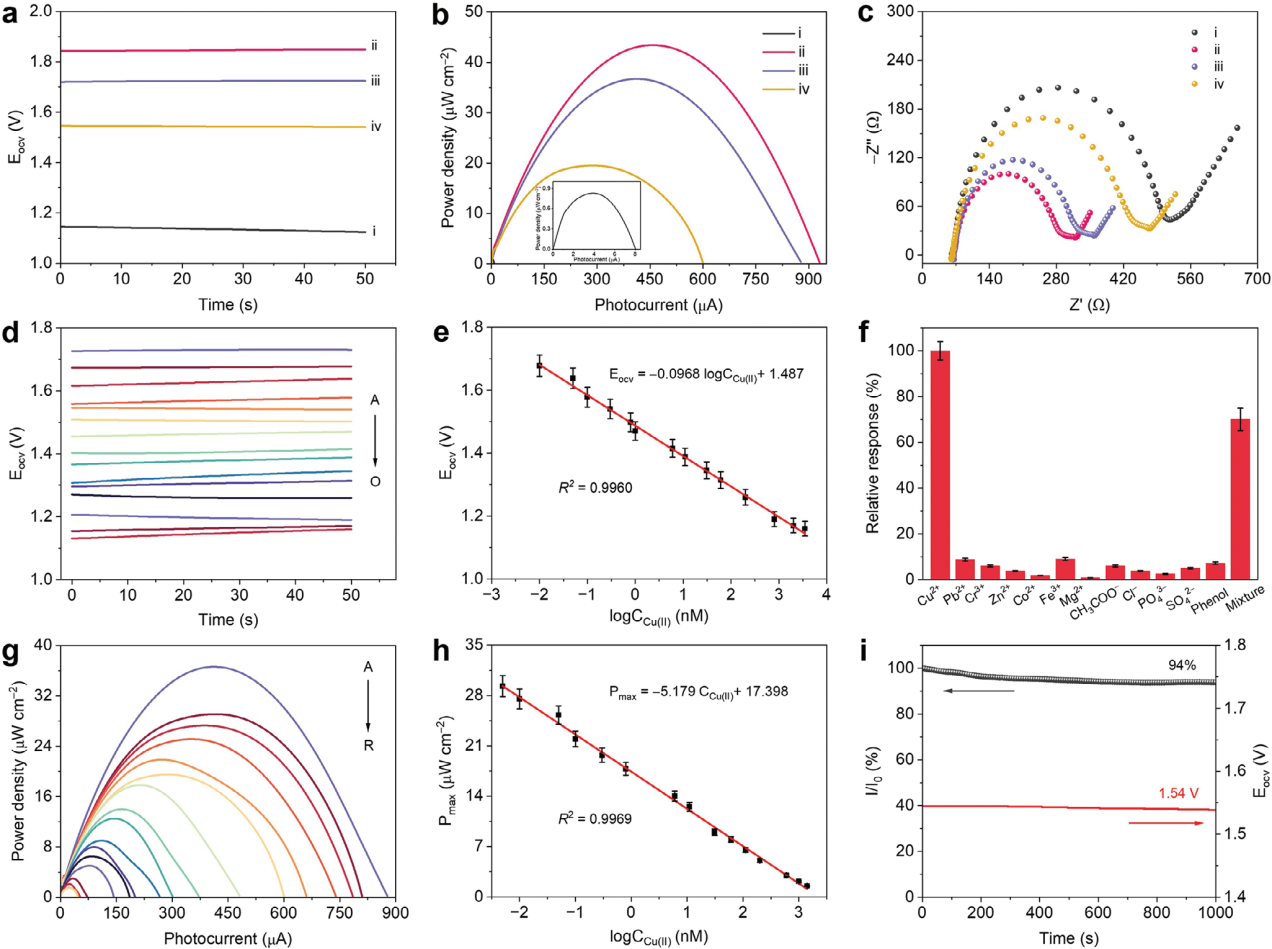
Detection performance. a) E_ocv_‐time curves, b) inset: *P–I* curve of bare ITO) *P–I* curves, and c) EIS spectra of the photoinduced ZAB‐SPES: (i) bare ITO, (ii) Co, S‐CN/ITO, (iii) apt/Co, S‐CN/ITO, (iv) apt/Co, S‐CN/ITO with Cu(II) incubated. d) E_ocv_‐time curves of the photoinduced ZAB‐SPES with different concentrations of Cu(II) (from A to O: 0, 0.01, 0.05, 0.1, 0.3, 0.8, 1, 6, 11, 31, 61, 200, 800, 2000, and 3500 nm). e) Linear relationship from 0.01 to 3500 nm. g) *P–I* curves of the photoinduced ZAB‐SPES with different concentrations of Cu(II) (from A to R: 0, 0.005, 0.01, 0.05, 0.1, 0.3, 0.8, 6, 11, 31, 61, 111, 200, 800, 1000, 1400, 2000, and 3500 nm). h) Linear relationship from 0.005 to 1400 nm. f) Selectivity and i) stability of the photoinduced ZAB‐SPES for Cu(II) detection. All error bars were obtained by three measurements.

Utilizing Cu(II) as the target model, Co, S‐CN was identified as the photocathode to construct the photoinduced ZAB‐SPES, utilizing a specific probe for the detection of Cu(II). Upon anchoring the aptamer molecules, both E_ocv_ (1.72 V, curve iii, **Figure** **5**a) and P_max_ (36.7 µW cm^−2^, Figure 5b) decreased, while the arc radius increased (Figure 5c). This phenomenon is attributed to steric hindrance caused by the non‐conductive probe molecules on the surface of the Co, S‐CN/ITO photocathode. Upon incubation with Cu(II) (0.3 nm), the E_ocv_ and P_max_ values further decreased, and the arc radius increased (curve iv). This indicates the inhibition of electron transfer from the Zn anode to the Co, S‐CN photocathode, which may impede the ORR, thus leading to the decreased E_ocv_ and P_max_ in the photoinduced ZAB. The photoinduced ZAB‐SPES with dual detection mode was developed for Cu(II) detection based on the Co, S‐CN photocathode. During the sensor construction process, the concentrations of Co, S‐CN dispersion and aptamer were optimized to enhance the sensitivity of Cu(II) detection (Figures S21 and S22, Supporting Information).

The analytical performance of the photoinduced ZAB‐SPES was evaluated for detecting Cu(II), including linearity, selectivity, stability, and reproducibility. As Cu(II) concentrations increased from 0 to 3500 nm, both E_ocv_ and P_max_ gradually decreased (Figure 5d,g; Figures S23 and S24, Supporting Information), indicating that the increased steric hindrance inhibits the ORR of the photocathode in the photoinduced ZAB owing to the formation of Cu(II)‐aptamer complexes. A linear correlation between the logarithm of Cu(II) concentration (logC_Cu(II)_) and E_ocv_ was expressed by the equation E_ocv_ = −0.0968logC_Cu(II)_ + 1.487 (Figure 5e), with a high correlation coefficient (*R^2^* = 0.9960). The linear range extended from 0.01 to 3500 nm, with a limit of detection (LOD, 3*S*/*N*) determined to be 4 pM. Within the linear range of 0.005 to 1400 nm, the relationship between P_max_ and logC_Cu(II)_ was described by the equation P_max_ = −5.179logC_Cu(II)_ + 17.398 (Figure 5h , *R^2^* = 0.9969), with a LOD of 2 pM, significantly lower than previously reported methods for Cu(II) (Table S8, Supporting Information). The selectivity of the sensor was evaluated against interfering ions and organic pollutants in water, such as Pb(II), Cr(III), Zn(II), Co(II), Fe(III), Mg(II), CH_3_COO^−^, Cl^−^, PO_4_^3−^, SO_4_^2−^, and phenol (at 100‐fold concentrations of Cu(II)), with a relative response of less than 20% in the presence of these interferences (Figure 5f). When mixed to the Cu(II) target, the sensor displayed a distinct response signal, demonstrating superior resistance to the interference. The enhanced selectivity is attributed to specific recognition between Cu(II)‐probe and Cu(II), indicating the potential of the photoinduced ZAB‐SPES platform for real water analysis. Stability was evaluated by incubating the constructed sensor with 0.3 nm Cu(II) under continuous irradiation and long‐term storage at 4 °C, respectively. The photocurrent signal (I) remained at 94% of the initial value (I_0_) after 1000 s of light illumination, with E_ocv_ barely changing (≈1.54 V, Figure 5i). After storage for 15 days, the E_ocv_ values slightly decreased (Figure S25, Supporting Information), suggesting highly stable detection for Cu(II). Reproducibility was evaluated with 60 nm Cu(II) incubated using six electrodes under the same modification, yielding nearly identical output signals (E_ocv_ and P_max_, Figure S26, Supporting Information). The practical application of the photoinduced ZAB‐SPES was evaluated in river water using the standard addition method. The river water (Yangtze River, Zhenjiang, China) was filtered to extract the supernatant, to which different concentrations of Cu(II) (0.1, 5, 50, and 1000 nm) were added. The recovery rates for the river water samples ranged from 94% to 102% with low relative standard deviation (RSD, Table S9, Supporting Information), indicating the feasibility of the photoinduced ZAB‐SPES for detecting Cu(II) in river water.

### 2.4 Portable Sensor Device

**Figure 6:**
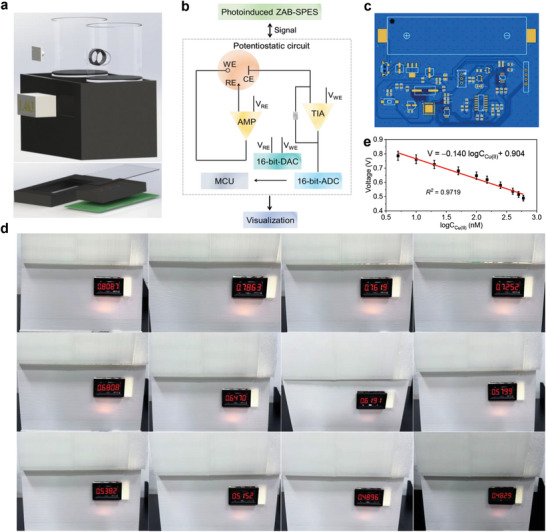
Device application. a) Overall construction diagram of the photoinduced ZAB‐SPES‐based device. b) Working principle of the detection sensor device. c) Photograph of the PCB. d) Photographs of the device with different concentrations of Cu(II): 0, 5, 10, 20, 50, 100, 150, 250, 400, 500, 600, and 700 nM. e) Linear relationship from 5 to 600 nm. All error bars were obtained by three measurements.

A portable device was designed based on the photoinduced ZAB‐SPES. This device (**Figure** **6**a) consisted of a shell (20 cm × 11 cm × 11 cm), a printed circuit board (PCB, 5.5 cm × 4 cm, Figure S27, Supporting Information), microelectronic components, a bulb (5 V, 3 W, white), a lithium‐ion battery (9 V), and a display terminal (LCD1602). The shell with a cover, 3D‐printed using polylactic acid, serves as a housing for the photoinduced ZAB‐SPES and as a dark box to prevent natural light from affecting the signal of the photoinduced ZAB‐SPES. The microelectronic components (Figure 6b,c) include resistors, capacitors, a microcontroller unit (MCU, STC15W408AS), an operational amplifier (AMP, LM358DR), a transresistance amplifier (TIA), and the display (Figure S28, Supporting Information). The bulb, functioning as a light source, was fixed externally to provide a stable input. The entire unit was powered by a lithium‐ion battery. The working principle of this detection device is as follows: the MCU controlled the digital‐to‐analog converter (DAC), converting the digital signal into an analog signal. The AMP and TIA formed the potentiostat module, which received the analog signal from the DAC for experimental measurements. The electrical signal generated by the photoinduced ZAB‐SPES was converted into a digital signal via an analog‐to‐digital converter (ADC) and visualized on the display. Similarly, the output voltage gradually decreased as Cu(II) concentrations increased from 0 to 700 nm (Figure 6d; Figure S29, Supporting Information). There was a linear relationship in the range of 5 ~ 600 nm, described by the equation V = −0.140 logC_Cu(II)_ + 0.904 (Figure 6e, *R^2^* = 0.9719), with a LOD of 1.7 nm. The feasibility of the device based on the photoinduced ZAB‐SPES was further evaluated for on‐site detection of lake water (Jiangsu University). Different concentrations of Cu(II) (10, 25, and 40 nM) were added to the lake water, and the voltage was recorded (Figure S30, Supporting Information). The recovery rates ranged from 95.2% to 106%, with RSD of 0.728%–2.82% (Table S10, Supporting Information), indicating that this device has the potential for on‐site and portable detecting Cu(II) in lake water.

## 3 Conclusion

A photoinduced ZAB‐SPES platform is proposed utilizing Co, S‐CN as the photocathode. Due to the synergistic effects of the C≡N functionalization and Co, S co‐doping, Co, S‐CN photocathode possesses excellent ORR performance, amplifying the E_ocv_ and P_max_ of the photoinduced ZAB. Based on the changes in E_ocv_ and P_max_, the photoinduced ZAB‐SPES demonstrates a low LOD (≈2 pM), high stability, and selectivity for heavy metal Cu(II) as the target model in the water environment. The photoinduced ZAB‐SPES‐based device is designed with a LOD of 1.7 nm, enabling on‐site detection of Cu(II) in lake water. This portable detection device in this study represents a significant advancement in the application of self‐powered electrochemical sensors based on photoinduced Zn‐air batteries.

## 4 Experimental Section

### Synthesis of Co, S‐CN

A molten salt‐assisted molecular assembly strategy was developed, followed by pyrolysis to synthesize Co, S‐CN (Figure 1a). L‐Cys (0.1 mmol) was dissolved in the mixture of ethanol (10 mL) and deionized water (20 mL), and stirred for 1 h until completely dissolved. After 30 min, the mixture of urea (2 g) and KHCO_3_ (0.1 g) was added and ball‐milled into the solution, which was stirred for 2 h. C_10_H_16_CoO_4_ (0.05 mmol) was added, and the resulting mixture was stirred for 12 h at room temperature. The fluffy precursor was obtained through vacuum freeze‐drying. The precursor was calcined for 2 h at 500 °C with a heating ramp of 5 °C min^−1^ under an argon atmosphere to obtain dark brown colored powder. The powder was dissolved into 0.1 m HCl solution and kept stirring for 2 h, followed by centrifugation, washing to neutral, and vacuum drying to denote Co, S‐CN. CN was synthesized using the same process without the addition of KHCO_3_, L‐Cys, and C_10_H_16_CoO_4_. Similarly, CCN was prepared without the addition of cobalt salt.

### Construction of the Photoinduced ZAB‐SPES

The photoinduced ZAB‐SPES platform was developed using a two‐chamber cell with a PPS membrane, where a Zn plate (3 cm × 2 cm) served as the anode, and Co, S‐CN acted as the photocathode (Figure 1b). Co, S‐CN material was modified on pretreated ITO glass. Cu(II)‐aptamer solution (0.5 µM) was added into the uniformly dispersed Co, S‐CN suspension (1 mg mL^−1^), subjected to agitation for 30 min, and this solution (200 µL) was deposited on the ITO conductive glass surface (1 cm × 2 cm). apt/Co, S‐CN/ITO photocathode was obtained after incubating at 4 °C for 10 h, followed by washing the electrode surface with PB (0.1 m, pH 7.0). Co, S‐CN/ITO photocathode was prepared using the same construction method, excluding the addition of the aptamer solution. Cu(II) solutions with different concentrations (20 µL) were coated onto apt/Co, S‐CN/ITO photocathode. After incubation for 40 min, the apt/Co, S‐CN/ITO with Cu(II) was constructed by washing with PB for the photoinduced ZAB‐SPES application. The modified photocathode and Zn plate anode were immersed in oxygen‐saturated PB (0.1 m, pH 7.0) and KOH (0.1 m, pH 13.6), where OH^−^ was transferred by anion exchange membrane. During the test, a highly uniformly intergated xenon light source (PLS‐FX300HU model, Beijing Perfectlight) with a power of 300 W was employed as the input excitation for the photocathode. The changes in E_ocv_ and P_max_ values were investigated through E_ocv_‐time and *V–I* curves, respectively. P_max_ values were determined by the equation of P_max_ = V × I/A, where V and I represent the voltage and photocurrent from the *V–I* curves, and A is the coverage area of the photocathode (A = 2 cm^2^).

### Statistical Analysis

All photoelectrochemical experiments were carried out three times in parallel. Data are presented as mean ± standard deviation. All error bars are equal to the standard deviation (*n* = 3).

## Conflict of Interest

The authors declare no conflict of interest.

## Supporting information



Supporting Information

## Data Availability

The data that support the findings of this study are available from the corresponding author upon reasonable request.
