# Supplementary material for: Photoinduced Zn‐Air Battery‐Assisted Self‐Powered Sensor Utilizing Cobalt and Sulfur Co‐Doped Carbon Nitride for Portable Detection Device
Source: Adv Sci (Weinh). 2024 Oct 24;11(46):2408293. doi: 10.1002/advs.202408293 (PMC11633469; doi:10.1002/advs.202408293)
Supplement: Supplementary file 1 — Supporting Information [file ADVS-11-2408293-s001.doc]

Supporting Information

Photoinduced Zn-Air Battery-Assisted Self-Powered Sensor Utilizing Cobalt and Sulfur Co-Doped Carbon Nitride for Portable Detection Device

Yun Chen, Yuhang Ge, Yuting Yan*, Li Xu, Xingwang Zhu, Pengcheng Yan, Penghui Ding*, Huaming Li, and Henan Li*

Y. Chen, Y. Ge, Y. Yan, L. Xu, P. Yan, H. Li, H. Li

School of Chemistry and Chemical Engineering, Institute for Energy Research, School of Agricultural Engineering

Jiangsu University

Zhenjiang 212013, China

E-mail: yanyuting@ujs.edu.cn; lhn@ujs.edu.cn

X. Zhu

School of Environmental Science and Engineering, College of Mechanical Engineering

Yangzhou University

Yangzhou 225002, China

P. Ding

Department of Science and Technology

Linköping University

Norrköping SE-601 74, Sweden

E-mail: penghui.ding@liu.se

**Materials**

Urea, L-Cysteine, KHCO_3_, (NH_4_)_3_PO_4_, NaH_2_PO_4_·2H_2_O, Na_2_HPO_4_·2H_2_O, K_3_[Fe(CN)_6_], K_4_[Fe(CN)_6_·3H_2_O], and KCl were obtained from Sinopharm (Shanghai, China). Cobalt(II) acetylacetonate (C_10_H_16_CoO_4_) was bought from Energy Chemical (Shanghai, China). Cr(CH_3_COO)_3_·6H_2_O was purchased from Macklin (Shanghai, China). A copper standard solution (Cu(II), 100 μg mL^−1^, 1% HNO_3_), Nafion (20 wt%), Pb(NO_3_)_2_, CoCl_2_·6H_2_O, ZnCl_2_, FeCl_3_, and phenol were bought from Sigma-Aldrich (Shanghai, China) were obtained from Aldrich (Shanghai, China). Cu(II)-aptamer with the sequence of 5'-ATCG CGAT ATTT TCTG TAGC GATT CTTG TTTG AGCG CTCG GTAC GAAC AGA-3' was bought from Sangon Biotech (Shanghai, China). Cd(NO_3_)_2_ and MgSO_4_ were purchased from Brilliant (Shanghai, China).

**Characterization**

Scanning electron microscope (SEM) images were recorded on JSM-7800F. Transmission electron microscope (TEM), high-resolution TEM (HRTEM), and energy-dispersive X-ray spectroscopy (EDX) mappings images were conducted on Talos F200S JEM-2010. Atomic force microscopy (AFM) images were characterized from Bruker Innova. Zeta potential of these samples was analyzed by Malvern NANOZS90. X-ray diffraction (XRD) was carried out on Shimadzu XRD-6100 with Bruker Cu Kα radiation. Attenuated total reflectance infrared spectroscopy (ATR-IR) were recorded by Nicolet iS50 with KBr as the blank. ^13^C solid-state nuclear magnetic resonance (NMR) spectra were carried out at AVANCE III 400 WB (4 mm probe) from Bruker. N_2_ absorption-desorption isotherms were conducted on a Brunauer-Emmett-Teller (BET) micropolitics from TriStar II 3020. Organic element analysis was recorded on FlashSmart from ThermoFisher. Inductively coupled plasma mass spectrometry (ICP-MS) was implemented from iCAP7400. X-ray photoelectron spectroscopy (XPS) was implemented at ESCALAB 250Xi. Thermogravimetric (TG) analysis was performed at the model of TG 209 from NETZSCH under N_2_ atmosphere with a heating rate of 10° min^−1^. Ultraviolet-visible (UV-Vis) absorption spectra were studied by UV-2600 from Shimadzu with BiSO_4_ as the blank. Ultraviolet photo-electron spectroscopy (UPS) spectra were performed by Nexsa G2 from Thermo Scientific. Electron paramagnetic resonance (EPR) spectra was employed at Bruker A300-10/12. Steady-state and time-resolved fluorescence (FL, America) spectra were measured by QuantaMaster^TM^40 at an excitation wavelength of 370 nm. Electron spin resonance spectroscopy (ESR) spectra were measured by JES X320 from JEOL.

**PEC measurements**

All photoelectrochemical (PEC) measurements were carried out by CHI660E electrochemical workstation from CH Instruments. Transient photocurrent, Mott-Schottky plots, electrochemical impedance spectroscopy (EIS), and linear sweep voltammetry (LSV) curves were measured by in phosphate buffer (PB, 0.1 M, pH 7.0) and a three-electrode system including indium tin oxide (ITO) modified electrode as the working electrode, platinum wire as the counter electrode, and Ag/AgCl electrode as the reference electrode. The excitation light source was a higly uniformly intergated xenon lamp (300 W, PLS-FX300HU) from Beijing Perfectlight. EIS spectra (0.01 Hz ~ 100 kHz) were measured in the impedance solution without or with light illumination. Mott-Schottky plots were measured in 0.5 M NaSO_4_ solution at frequencies of 1.5, 2.0, and 3.0 kHz, where glassy carbon electrode (GCE) acted as the working electrode and modified with different suspension (5 μL, 5 mg mL^−1^). Open circuit voltage (E_ocv_) and the maximum power density (P_max_) were acquired by a two-chamber cell with a polyphenylene sulfide (PPS) membrane, in which a Zn plate (3 cm × 2 cm) and the modified ITO electrode served as the anode and photocathode, respectively.

**Scheme S1.** Schematic illustration for the synthetic structure for a) CN, b) CCN, and c) Co, S-CN.


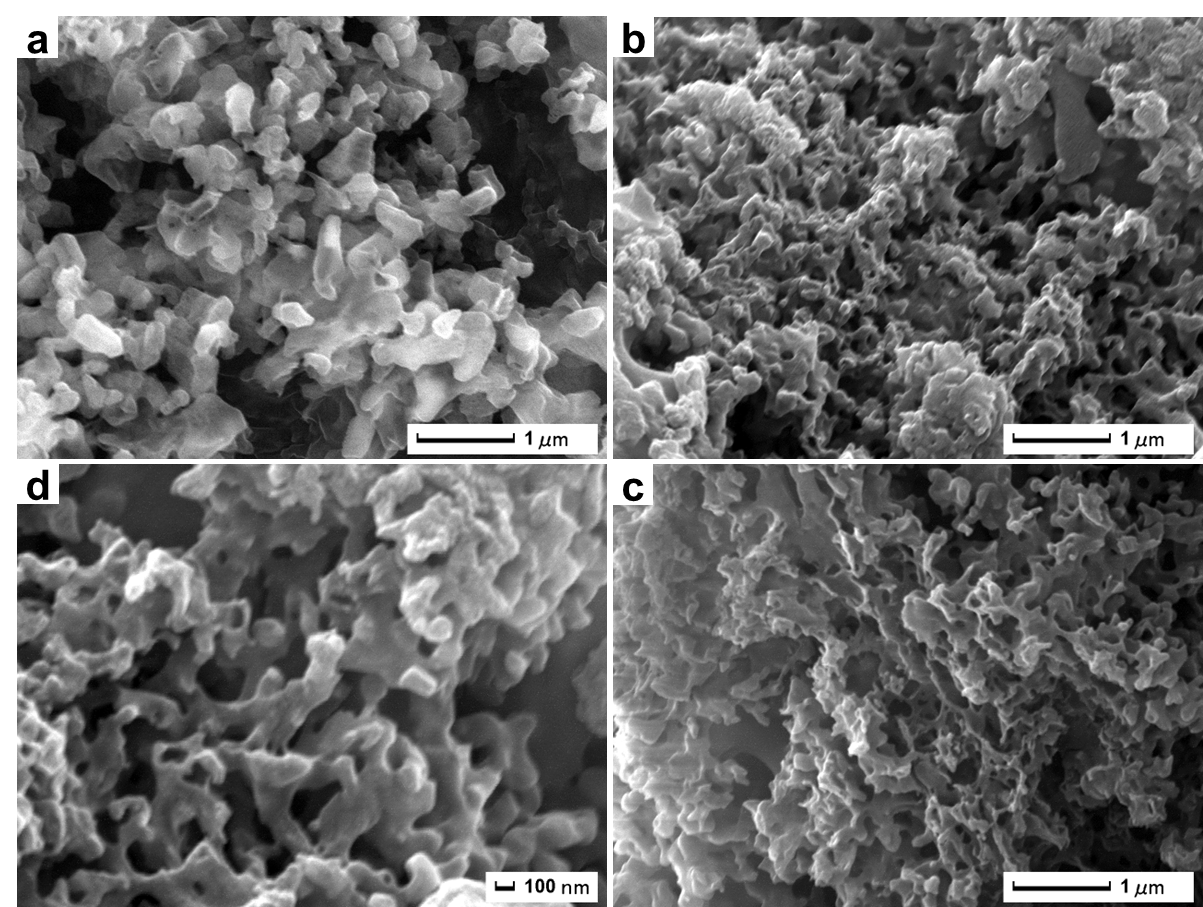


**Figure S1.** SEM images of a) CN, b and c) CCN, and d) Co, S-CN.


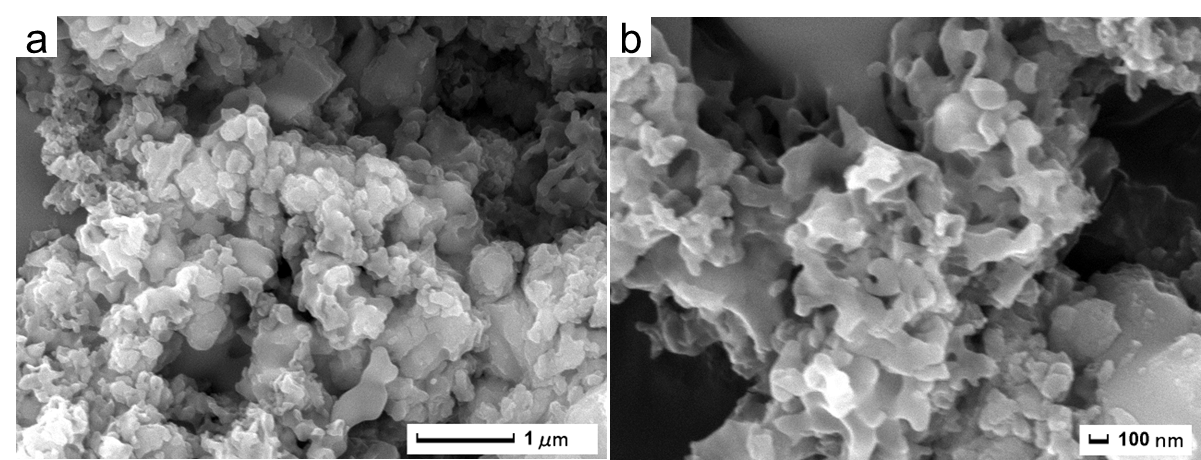


**Figure S2.** SEM images of CN-C.


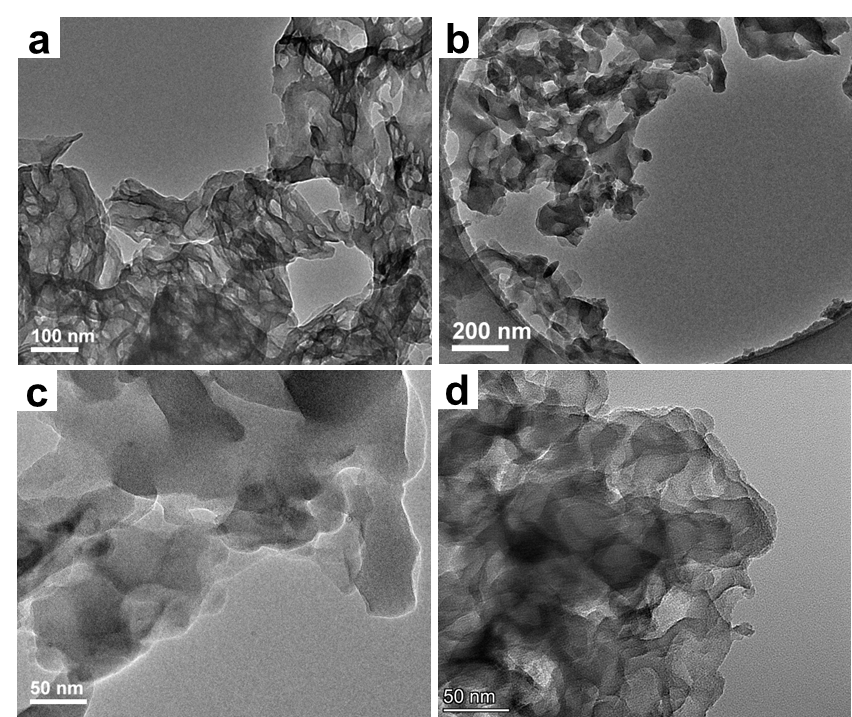


**Figure S3.** TEM images of a) CN, b and c) CCN, and d) Co, S-CN.

**Figure S4.** AFM image and corresponding height of CCN.

**Figure S5.** a and b) HRTEM images of Co, S-CN.

**Figure S6.** EDX mapping images of C, N, O, Co, K, and S elements for Co, S-CN.

**Figure S7.** Zeta potential of CN, CCN, and Co, S-CN.

**Figure S8.** TG curves of CN, CCN, and Co, S-CN.

**Figure S9.** Survey XPS spectra of CN, CCN, and Co, S-CN.

**Figure S10.** High-resolution a) C 1s, b) N 1s, and c) O 1s XPS spectra of CN, CCN, and Co, S-CN. d) High-resolution K 2p XPS spectra of CCN and Co, S-CN.

**Figure S11.** VB-XPS spectra of CN, CCN, and Co, S-CN.

**Figure S12.** UPS spectra of a) CN and b) CCN.

**Figure S13.** Mott-Schottky plots of a) CN and b) CCN. All data were obtained by three measurements.

**Figure S14.** Photocurrent response of Co, S-CN with different L-Cysteine content. All data were obtained by three measurements.

**Figure S15.** Photocurrent response of of Co, S-CN with different cobalt salts content. All data were obtained by three measurements.

**Figure S16.** Photocurrent response with different fixed area of Co, S-CN under the same load capacity. All data were obtained by three measurements.

**Figure S17.** LSV curves of Co, S-CN/ITO electrode with light illumination in PB under the saturated N_2_ and O_2_ conditions. All data were obtained by three measurements.

**Figure S18.** ESR spectra of DMPO-superoxide radical with and without illumination for a) CN, b) CCN, and c) Co, S-CN (c).

**Figure S19.** ESR spectra of DMPO-hydroxyl radical with and without illumination for a) CN, b) CCN (b), and c) Co, S-CN.

**Figure S20.** E_ocv_-time curves of the ZAB-SPES system based on Co, S-CN photocathode without and with light illumination. All data were obtained by three measurements.

**Figure S21.** E_ocv_ values of Co, S-CN dispersion concentration. All error bars were obtained by three measurements.

**Figure S22.** Effect of aptamer concentration of the photoinduced ZAB-SPES system for Cu(II) detection. All error bars were obtained by three measurements.

**Figure S23.** E_ocv_ values of the photoinduced ZAB-SPES with different concentrations of Cu(II): 0, 0.01, 0.05, 0.1, 0.3, 0.8, 1, 6, 11, 31, 61, 200, 800, 2000, and 3500 nM. All error bars were obtained by three measurements.

**Figure S24.** P_max_ values of the photoinduced ZAB-SPES with different concentrations of Cu(II): 0, 0.005, 0.01, 0.05, 0.1, 0.3, 0.8, 6, 11, 31, 61, 111, 200, 800, 1000, 1400, 2000, and 3500 nM. All error bars were obtained by three measurements.

**Figure S25.** Long-term stability of the photoinduced ZAB-SPES with Cu(II) incubated. All error bars were obtained by three measurements.

**Figure S26.** Reproducibility of the photoinduced ZAB-SPES with Cu(II) incubated. All error bars were obtained by three measurements.


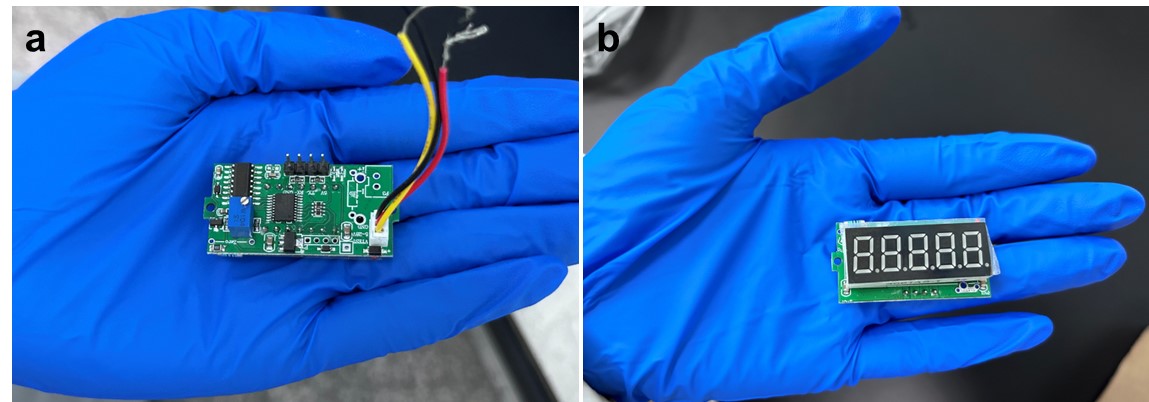


**Figure S27.** Photographs of the PCB and the display terminal.


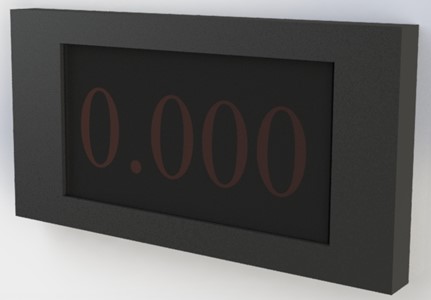


**Figure S28.** Diagram of a display terminal.

**Figure S29.** Nonlinear curve of the device between different concentrations of Cu(II) and voltage: 0, 5, 10, 20, 50, 100, 150, 250, 400, 500, 600, and 700 nM. All error bars were obtained by three measurements.


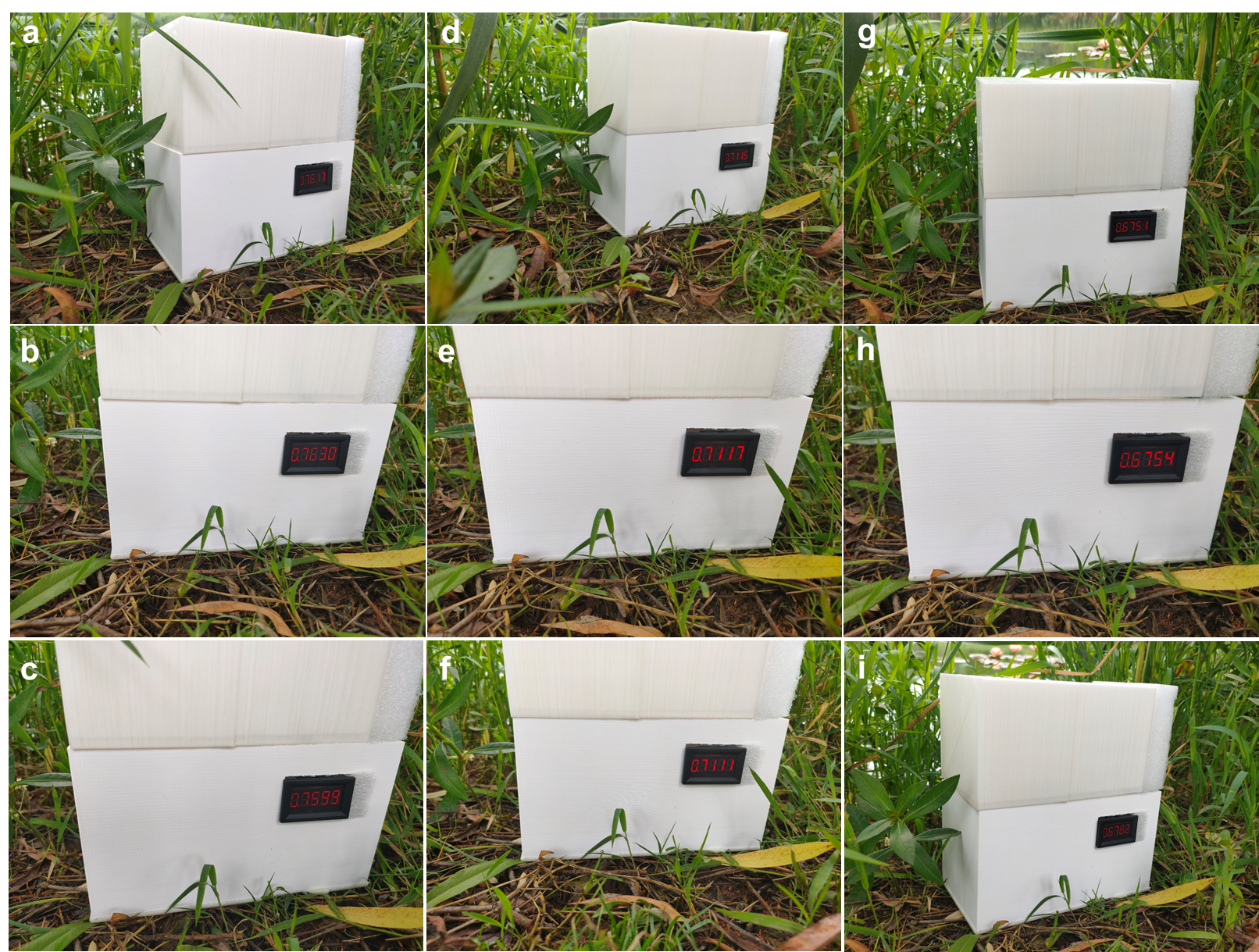


**Figure S30.** Photographs of the photoinduced ZAB-SPES-based device for on-site detection in lake water with different concentrations of Cu(II): (a-c) 10 nM, (d-f) 25 nM, (g-i) 40 nM. All data were obtained by three measurements.

**Table S1.** Specific surface area and pore volume of CN, CCN, and Co, S-CN.

| **Sample** | **S_BET_ (m^2^ g^−1^)** | **Pore volume (cm^3^ g^−1^)** |
| --- | --- | --- |
| CN | 22.71 | 0.021 |
| CCN | 9.64 | 0.011 |
| Co, S-CN | 41.68 | 0.061 |

**Table S2.** Organic element analysis of CN, CCN, and Co, S-CN.

| **Sample** | **C (%)** | **N (%)** | **C/N** |
| --- | --- | --- | --- |
| CN | 33.22 | 60.53 | 0.549 |
| CCN | 26.93 | 44.48 | 0.605 |
| Co, S-CN | 26.85 | 43.77 | 0.613 |

**Table S3.** Element content of Co, S-CN by ICP-MS spectra.

| **Element** | **Content (wt%)** |
| --- | --- |
| Co | 0.60 |

**Table S4.** Area ratio of C−C or C=C to N−C=N from C 1s XPS spectra of CN, CCN, and Co, S-CN.

| **Sample** | **C−C or C=C/N−C=N** |
| --- | --- |
| CN | 0.177 |
| CCN | 0.379 |
| Co, S-CN | 0.290 |

**Table S5.** Electronic band structure of CN, CCN, and Co, S-CN by UV-Vis absorption and UPS spectra.

| **Sample** | ***E*_g_ (eV)** | ***E*_F_ (eV)** | ***E*_VB_ (eV *vs*. vac)** | ***E*_CB_ (eV vs vac)** |
| --- | --- | --- | --- | --- |
| CN | 2.77 | −4.94 | −6.11 | −3.34 |
| CCN | 1.94 | −5.22 | −6.15 | −4.21 |
| Co, S-CN | 2.02 | −5.06 | −5.98 | −3.96 |

**Table S6.** Average lifetime for carriers of CN, CCN, and Co, S-CN from transient FL spectra.

| **Sample** | ***τ*_av_ (ns)** |
| --- | --- |
| CN | 5.26 |
| CCN | 2.02 |
| Co, S-CN | 1.03 |

**Table S7.** Comparison with other ZAB-SPES.

| **Material** | **E_ocv_ (V)** | **P_max_ (μW cm^−2^)** | **Target** | **LOD** | **Ref.** |
| --- | --- | --- | --- | --- | --- |
| CsPbBr_3_@COF | 1.556 | - | PTP1B | 0.032 pM | [1] |
| MOFs@GOX | - | 22.8 | miRNA let-7a | 1.38 fM | [2] |
| Co-NC@mHCSs | 1.411 | 1.784×10^5^ | SARS-CoV-2 S-gene | 0.45 fg mL^–1^ | [3] |
| AgBr/CuBi_2_O_4_ | 1.99 | - | Gallic acid | 0.87 μM | [4] |
| Co_3_O_4_/  LIG | 1.39 | - | Pulse signal | - | [5] |
| MnOx@C-700 | 1.34 | 2.22×10^3^ | Diethylstilbestrol | 0.08 pg mL^–1^ | [6] |
| pTTh/Au NPs | 1.5 | - | Glucose | 73.7 nM | [7] |
| WO_3_·H_2_O | 1.22 | 42 | Microcystin | 1.31 fM | [8] |
| Co, S-CN | 1.85 | 43.5 | Cu(II) | 2 pM | This work |

**Table S8.** Comparison with other sensors for Cu(II) detection.

| **Detection method** | **Detection range (nM)** | **LOD (nM)** | **Reference** |
| --- | --- | --- | --- |
| Electrochemistry | 4.0×10^2^-1.3×10^4^ | 50 | [9] |
| Fluorometry | 5-2×10^2^ | 1.7 | [10] |
| Electrochemistry | 1.0×10^2^-3.5×10^3^ | 7 | [11] |
| Electrochemical stripping | 20-1.0×10^4^ | 1.8 | [12] |
| Photoelectrochemistry | 0-6.5×10^7^ | 6.9 | [13] |
| Colorimetric and fluorescence chemosensor | - | 20.4 | [14] |
| Photoinduced ZAB-SPES | 5×10^−3^-1.4×10^3^ | 2×10^−3^ | This work |

**Table S9.** This photoinduced ZAB-SPES for Cu(II) detection in actual river water samples.

| **Sample** | **Spiked (nM)** | **Found (nM)** | **Recovery (%)** | **RSD (%, n = 3)** |
| --- | --- | --- | --- | --- |
| 1 | 0.1000 | 0.09400 | 94.00 | 6.00 |
| 2 | 5.000 | 4.950 | 99.00 | 2.11 |
| 3 | 50.00 | 51.00 | 102.0 | 4.74 |
| 4 | 1000 | 992.0 | 99.20 | 2.19 |

**Table S10.** This portable device for Cu(II) detection in lake water.

| **Sample** | **Spiked (nM)** | **Found (nM)** | **Recovery (%)** | **RSD (%, n = 3)** |
| --- | --- | --- | --- | --- |
| 1 | 10.0 | 10.5 | 105 | 2.52 |
| 2 | 25.0 | 23.8 | 95.2 | 0.728 |
| 3 | 40.0 | 42.4 | 106 | 2.82 |

**References**

1. K. Xiao, R. Zhu, C. Du, H. Zheng, X. Zhang, J. Chen, *Anal*. *Chem*. **2022**, *94*, 9844-9850.
2. Y. Jin, Z. Wu, L. Li, R. Yan, J. Zhu, W. Wen, X. Zhang, S. Wang, *Anal*. *Chem*. **2022**, *94*, 14368-14376.
3. X. Tang, Y. Wang, Z. Zhang, M. Xu, Z. Tao, S. Li, J. Liu, Z. Peng, C. Guo, L. He, R. Wang, M. Du, Z. Zhang, S. H. Kim, *Nano Energy* **2024**, *127*, 109713.
4. Y. Yang, X. Du, D. Jiang, X. Shan, W. Wang, H. Shiigi, Z. Chen, *Sens*. *Actuators B* **2023**, *393*, 134302.
5. X. Chen, Z. Hou, G. Li, W. Yu, Y. Xue, G. Niu, M. Xin, L. Yang, C. Meng, S. Guo, *Nano Energy* **2022**, *101*, 107606.
6. C. Guo, Y. Ruan, S. Zhang, L. Kan, H. Bian, F. Rong, L. He, D. Li, M. Du, Z. Zhang, *Chem*. *Eng*. *J*. **2023**, *466*, 143033.
7. J. Zhu, W. Nie, Q. Wang, W. Wen, X. Zhang, F. Li, S. Wang, *Chem*. *Commun*. **2020**, *56*, 5739-5742.
8. Q. Wang, D. Jiang, X. Du, X. Shan, W. Wang, H. Shiigi, Z. Chen, *Analyst* **2024**, *149*, 2291-2298.
9. Z. Xu, Q. Meng, Q. Cao, Y. Xiao, H. Liu, G. Han, S. Wei, J. Yan, L. Wu, *Anal*. *Chem*. **2020**, *92*, 2201-2206.
10. Y. Fan, H. Xing, Y. Xue, C. Peng, J. Li, E. Wang, *Anal*. *Chem*. **2020**, *92*, 16066-16071.
11. M. Lu, Y. Deng, Y. Luo, J. Lv, T. Li, J. Xu, S. W. Chen, J. Wang, *Anal*. *Chem*. **2019**, *91*, 888-895.
12. Y. Xia, Y. Zhao, F. Ai, Y. Yi, T. Liu, H. Lin, G. Zhu, *J. Hazard*. *Mater*. **2022**, *425*, 127974.
13. C. Zhai, L. Miao, Y. Zhang, L. Zhang, H. Li, S. Zhang, *Chem. Eng. J.* **2022**, *431*, 134107.
14. D. Aydin, S. N. Karuk Elmas, F. N. Arslan, *Food Chem*. **2023**, *402*, 134439.
